# Supplementary material for: Visualizing symmetry-breaking electronic orders in epitaxial Kagome magnet FeSn films
Source: Nat Commun. 2023 Oct 4;14:6167. doi: 10.1038/s41467-023-41831-4 (PMC10550950; doi:10.1038/s41467-023-41831-4)
Supplement: Supplementary file 1 — Supplementary Information [file 41467_2023_41831_MOESM1_ESM.pdf]

Supplementary Information

Visualizing symmetry-breaking electronic orders in epitaxial Kagome magnet FeSn films

Huimin Zhang[1,2], Basu Dev Oli[1], Qiang Zou[1], Xu Guo[3], Zhengfei Wang[3] and Lian Li[1★]

[1]Department of Physics and Astronomy, West Virginia University, Morgantown, WV26506, USA

[2]State Key Laboratory of Structural Analysis, Optimization and CAE Software for Industrial Equipment, Dalian University of Technology, Dalian 116024, China

[3]Hefei National Research Center for Physical Sciences at the Microscale, CAS Key Laboratory of Strongly-Coupled Quantum Matter Physics, Department of Physics, Hefei National Laboratory, University of Science and Technology of China, Hefei, Anhui 230026, China

★Correspondence to: lian.li@mail.wvu.edu

This file includes Figures S1-19, including simulated STM images of the Sn- and Fe_3Sn-terminated FeSn by DFT calculations (Figs. S6&7)

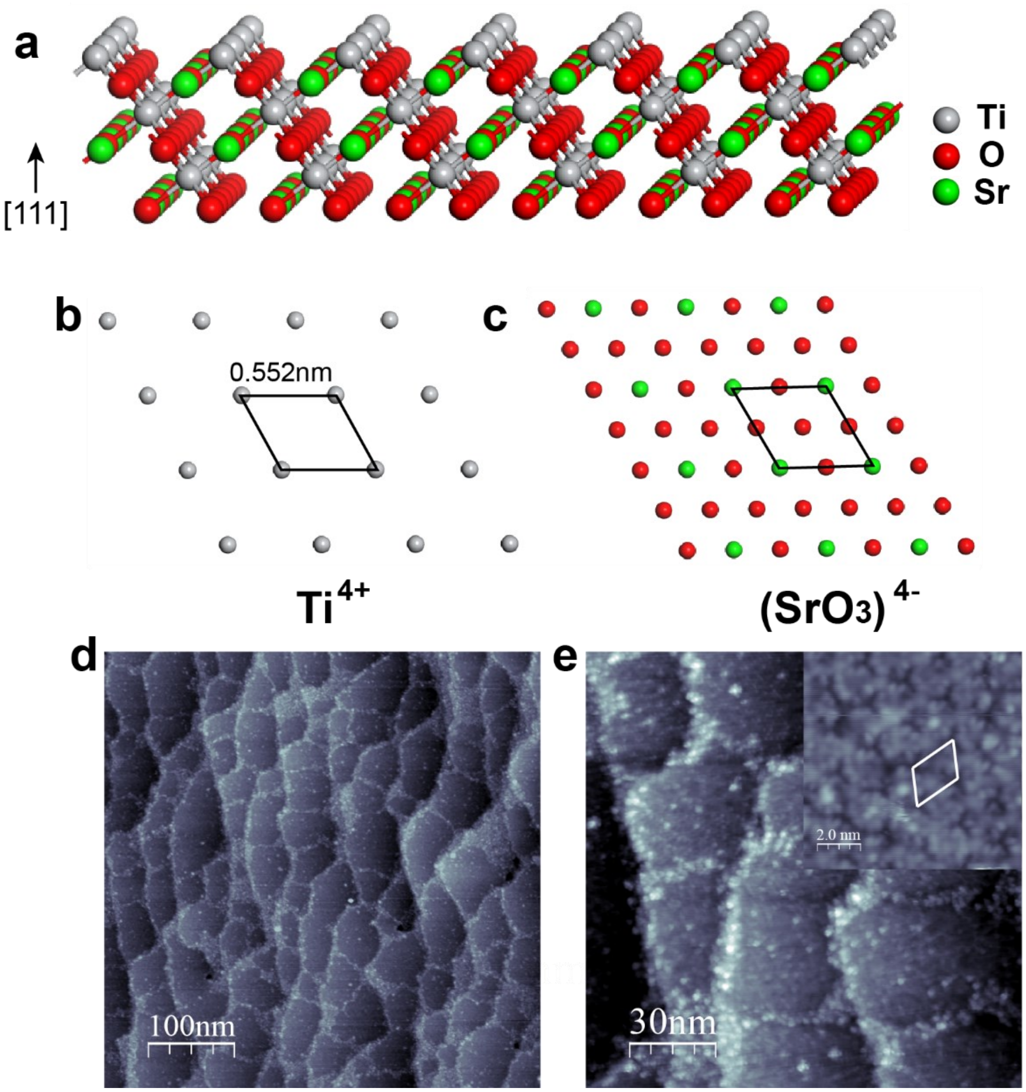

Figure S1. STM imaging of the $SrTiO_3$(111) substrate. a, Ball-and-stick model of the $SrTiO_3$(111) surface from side-view. **b**-**c**, Top-view of $(Ti)^{4+}$ and $(SrO_3)^{4-}$ planes, respectively. **d**, Topographic STM image of thermally treated $SrTiO_3$(111) substrate. Setpoint: V = 3.0 V, I = 30 pA. **e**, Zoom-in image revealing domain structure with a (4 × 4) reconstruction (Inset). Setpoint: V = 3.0 V, I = 30 pA and V = 1.0 V, I = 500 pA (inset).

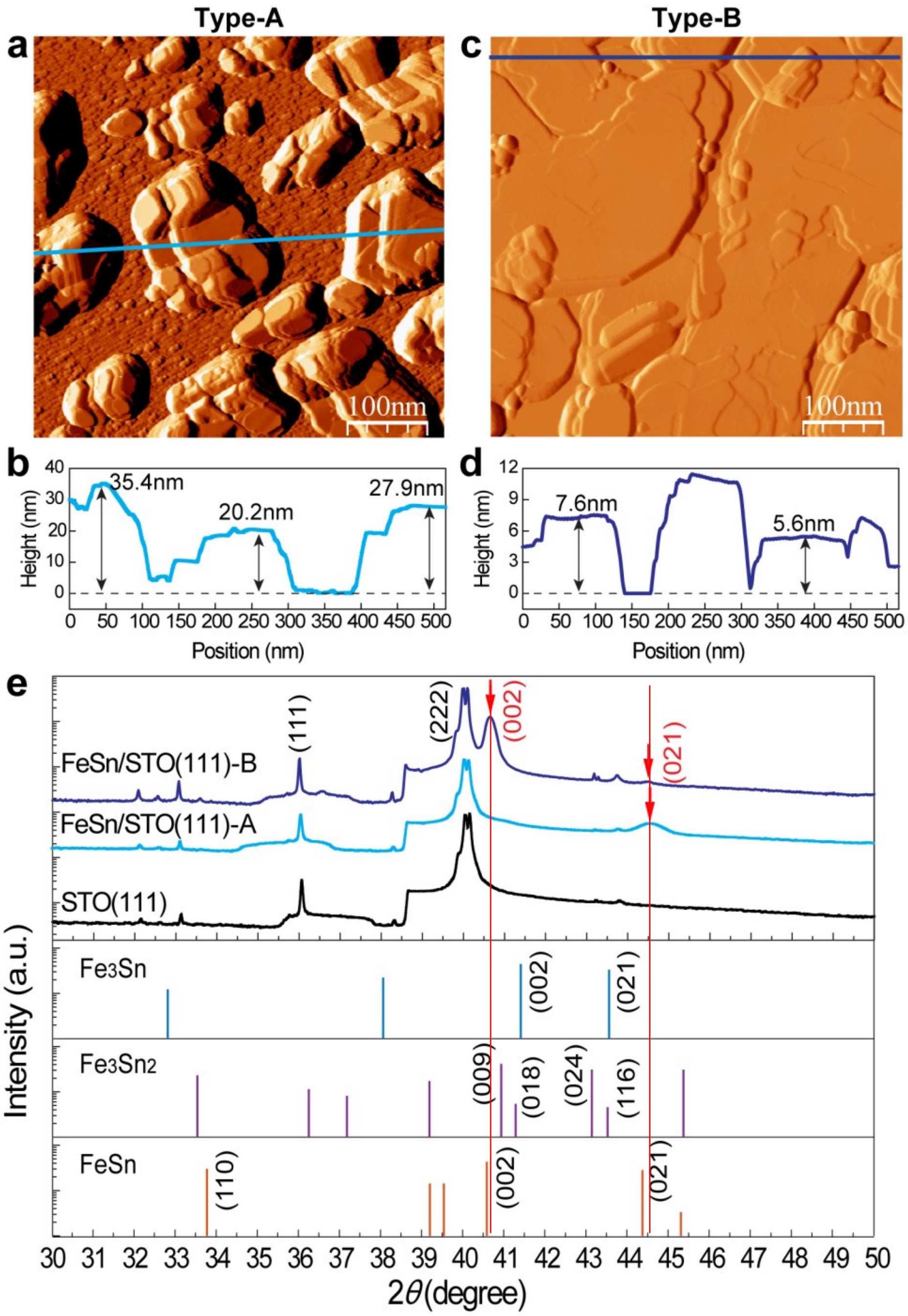

Figure S2. X-ray diffraction (XRD) of the FeSn/SrTiO$_3$(111) film. **a**, Topographic STM image of Type-A FeSn/STO(111) film. Setpoint: V = 3.0 V, I = 10 pA. **b**, Line profile along the cyan line in **a**. **c**, Topographic STM image of a Type-B FeSn/STO(111) film. Setpoint: V = 1.0 V, I = 100 pA. **d**, Line profile along the blue line in **c**. **e**, XRD patterns for FeSn films on STO(111) substrate. Reference data are Fe$_3$Sn (ICSD 24569), Fe$_3$Sn$_2$ (ICSD 71), FeSn (ICSD 103634), and α-Fe (ICSD 53451) from the Inorganic Crystal Structure Database. Here, we compare the XRD pattern with the references.

The peaks at $2\theta = 40.52^\circ$ *and* 44.58° are indexed as FeSn (002) and (012), respectively, indicating the phase is FeSn. Type A films were grown in an MBE system that is interfaced with Unisoku low-temperature STM, where images, dI/dV spectra, and dI/dV maps were taken at 4.5 K. Type B films were grown in a separate standing-alone MBE chamber. Note that the FeSn (002) and (021) peaks are slightly shifted to higher values (marked by the two red lines), indicating a smaller lattice constant in the *c*-direction.

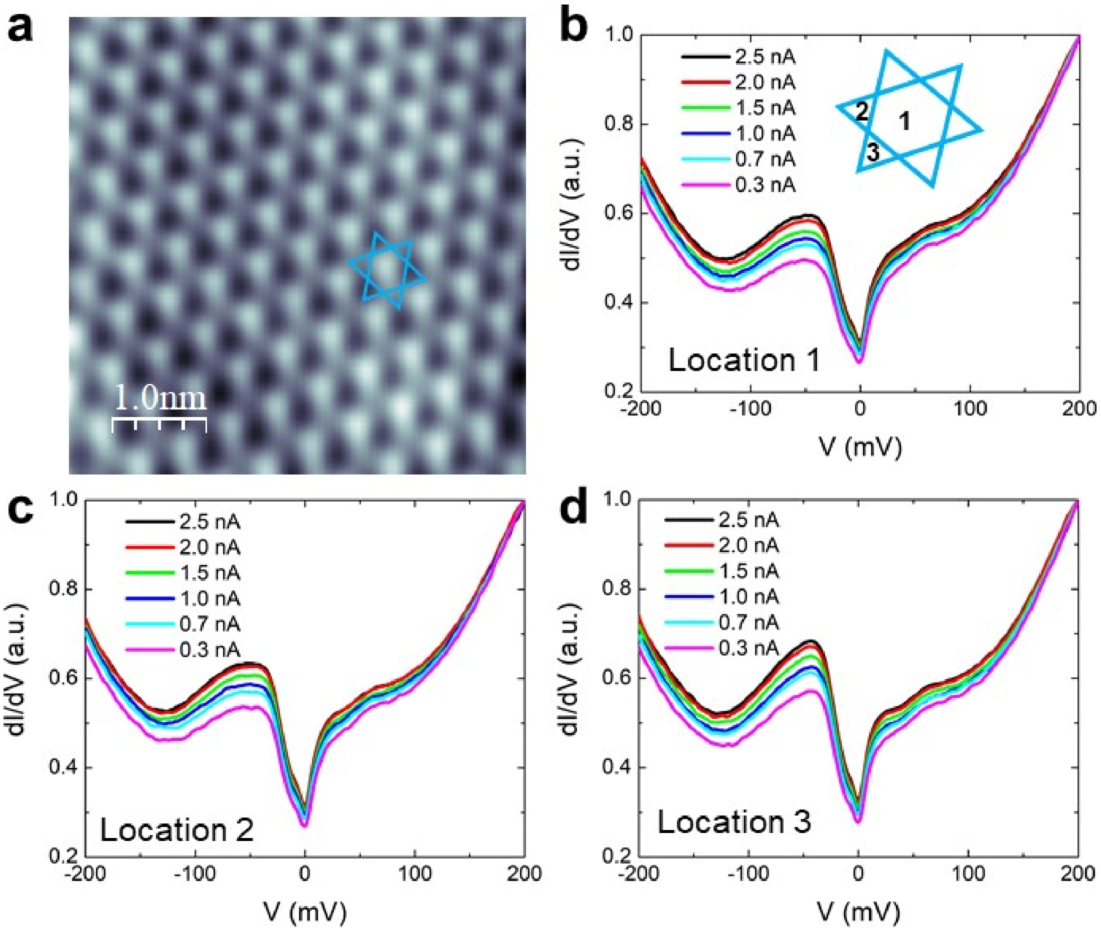

Figure S3. Setpoint-dependent dI/dV spectra. a, Topographic STM image of Fe_3Sn Kagome layer. Setpoint: *V* = 200 mV. **b-c**, dI/dV spectra taken at three typical locations labeled as 1, 2, and 3 in the inset of **b**. The tunneling current is varied from 0.3 nA to 2.5 nA.

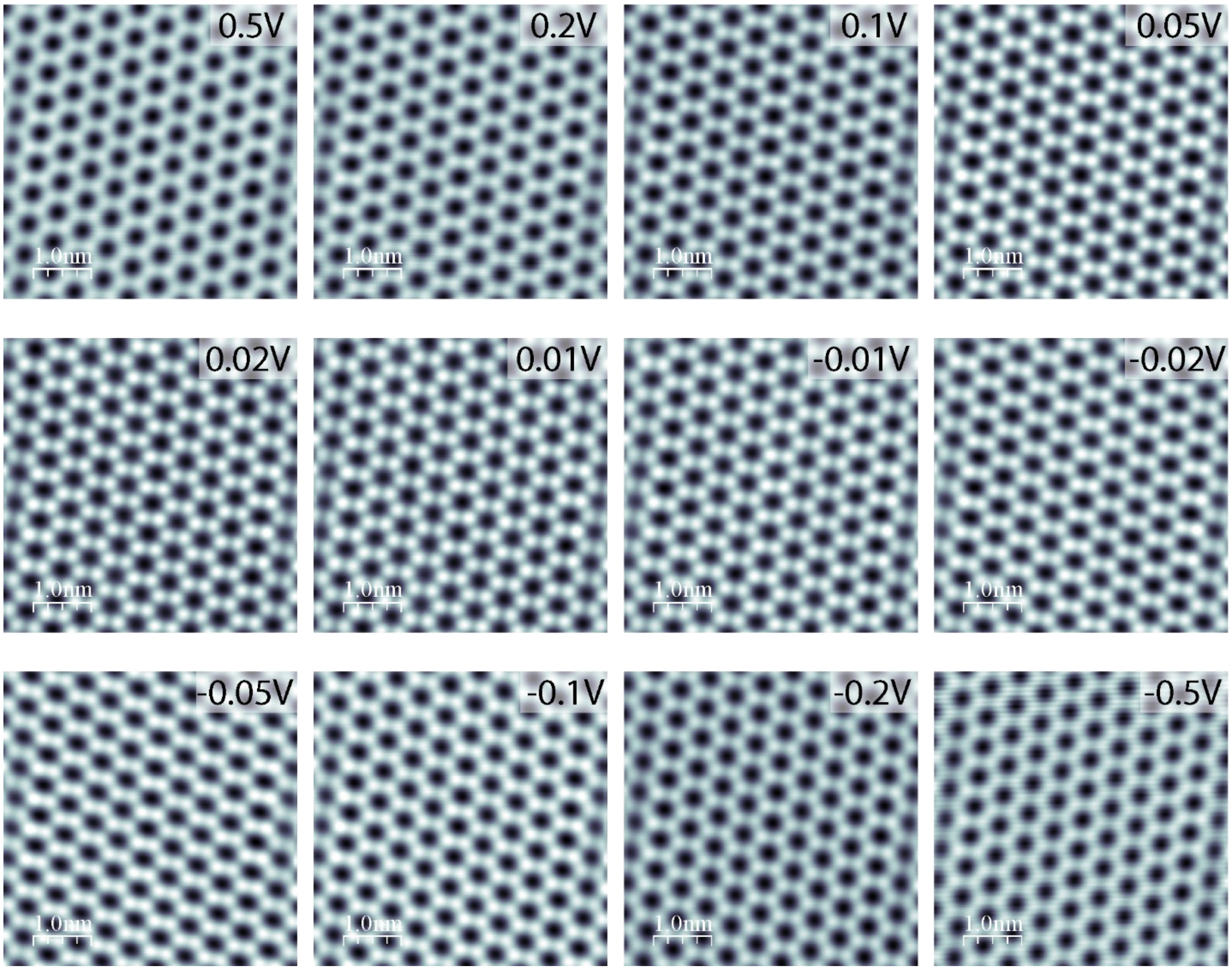

Figure S4. Bias-dependent STM imaging of the Sn honeycomb lattice. STM images of the Sn honeycomb structure on FeSn film's Sn-termination at the specified bias voltage. Setpoint: *I* = 5.0 nA.

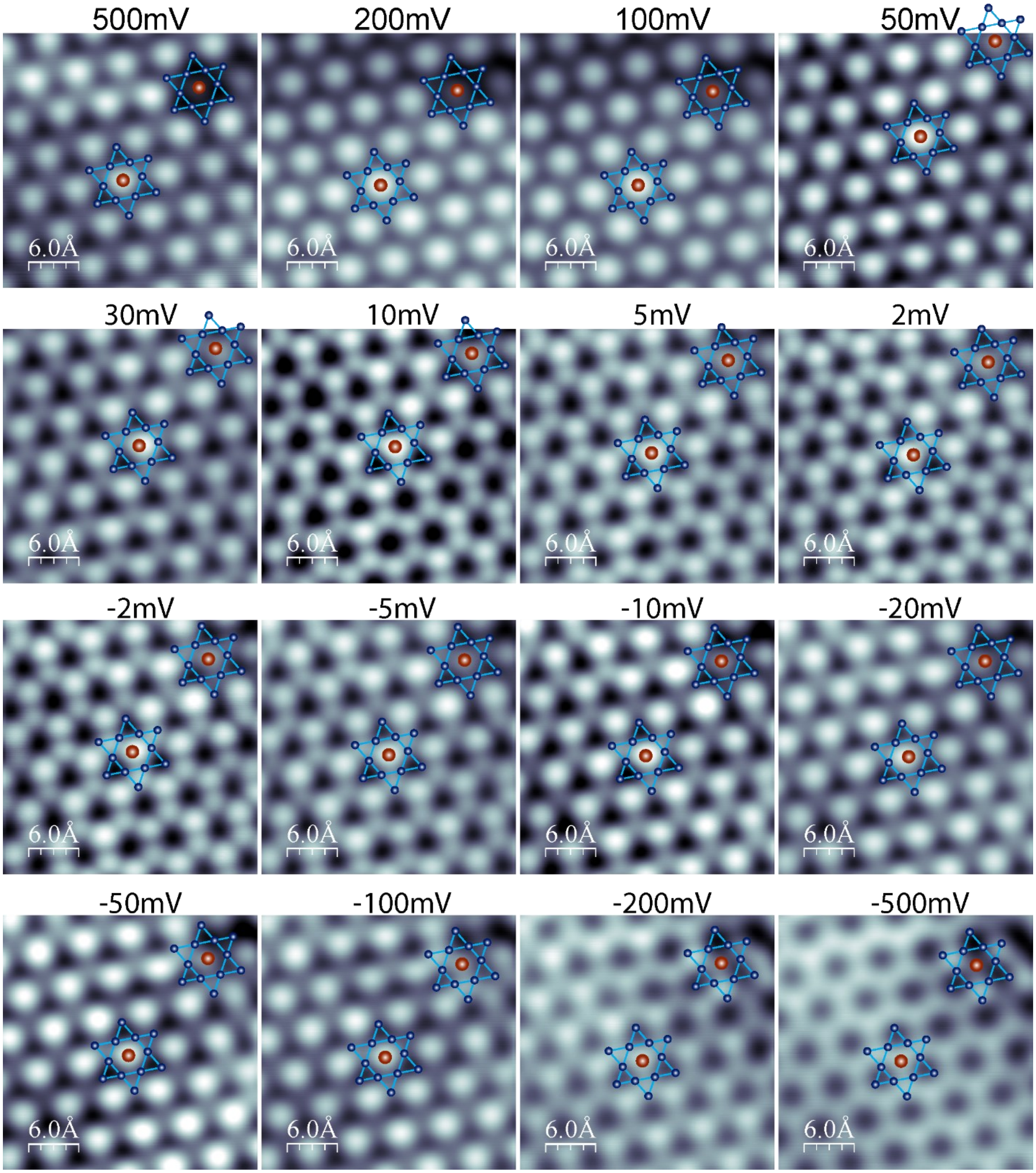

Figure S5. Bias-dependent STM imaging of the Fe_3Sn Kagome lattice. STM images of the Fe_3Sn-termination at the bias voltage specified. Setpoint: I = 3.0 nA. The ball-and-stick model of the Kagome lattice is overlaid on the surface. An Sn vacancy at the up-right corner is used as a reference to assign the atomic lattice.

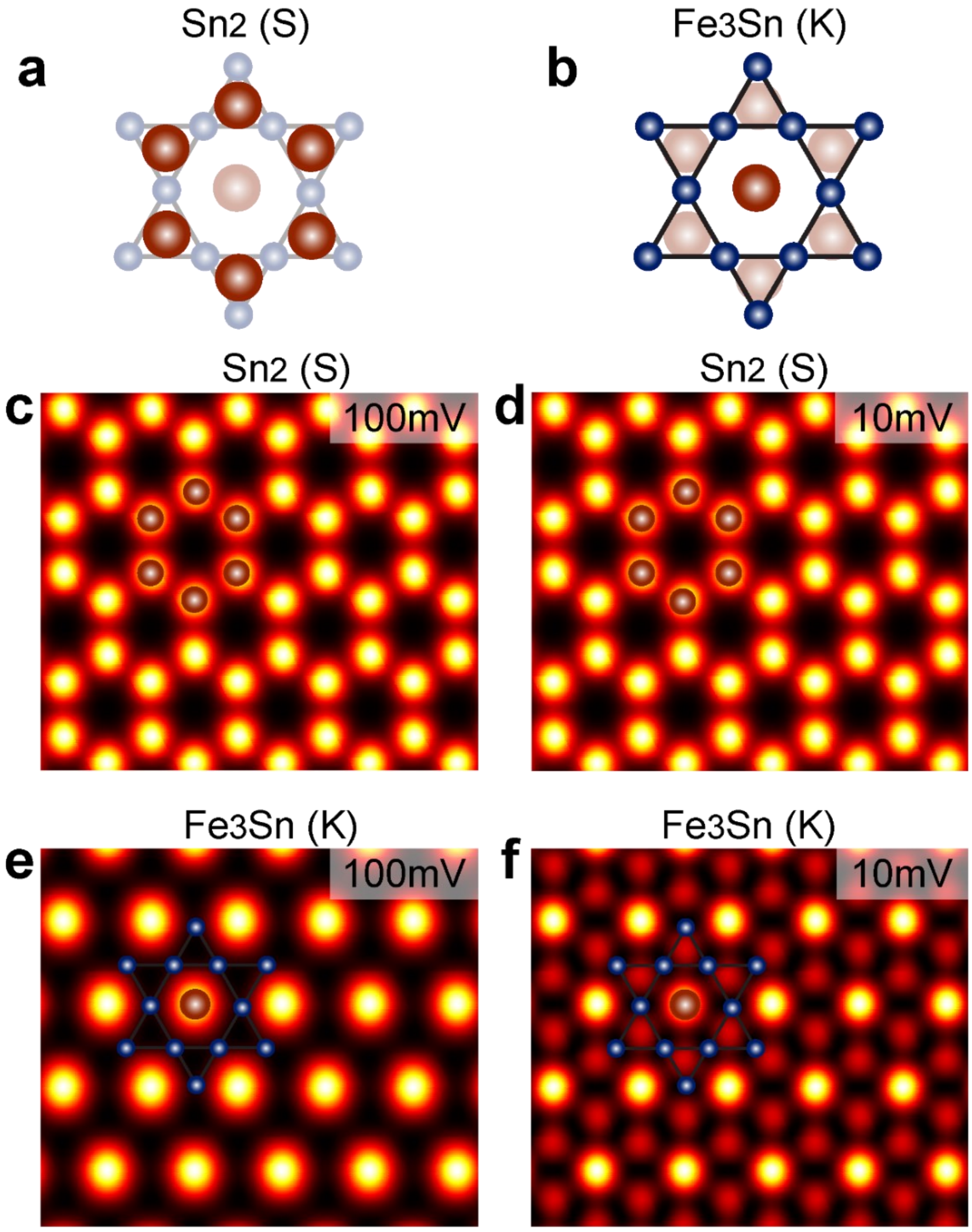

Figure S6. Simulated STM imaging of the Sn_2 (S) and Fe_3Sn (K) terminations by DFT. a, Schematic Sn_2 (S) layer with underlying K layer. The K layer is demonstrated in transparency. **b**, Schematic Fe_3Sn (K) layer with underlying S layer. **c**-**d**, Simulation of Sn_2 (K) layer under different bias. **e**-**f**, Simulation of Fe_3Sn (K) layer under different bias. The band alignment is based on the comparison with STM images and DFT simulation.

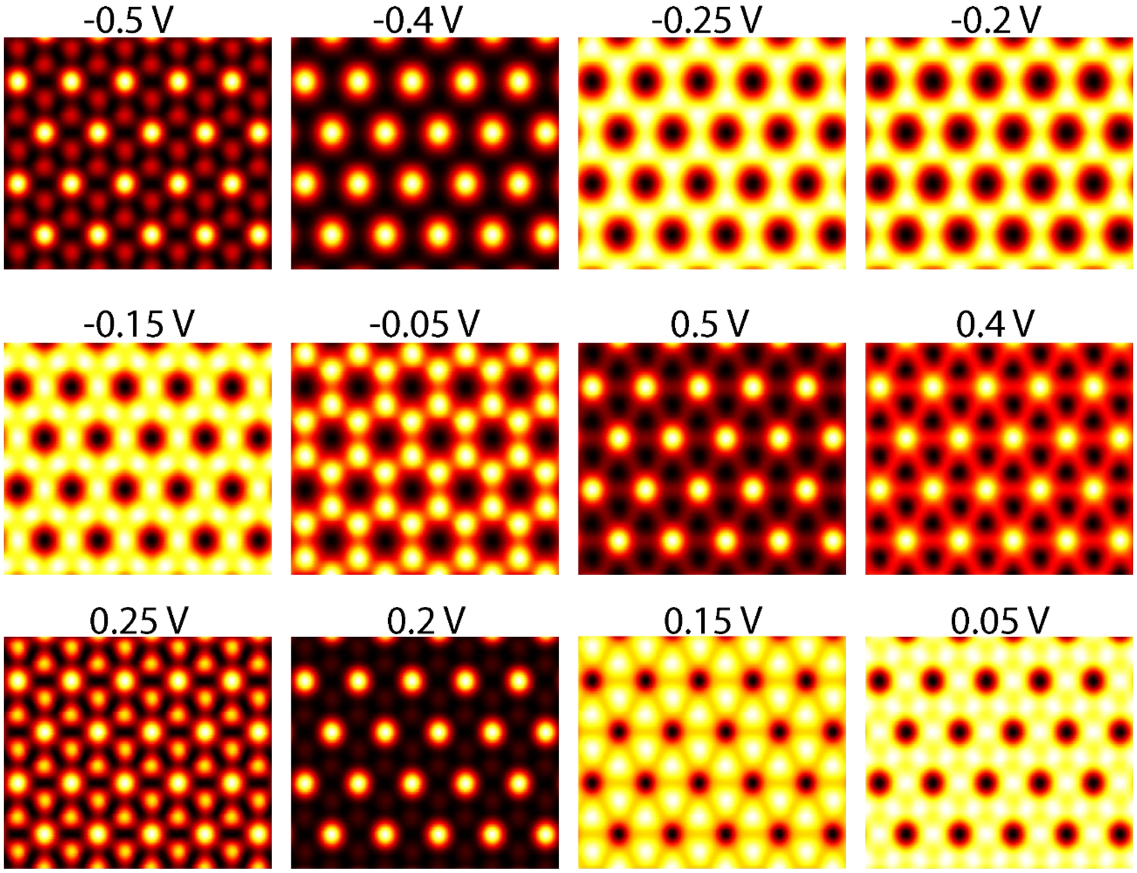

Figure S7. Simulation of the Fe_3Sn termination (K layer) at energies marked by DFT.

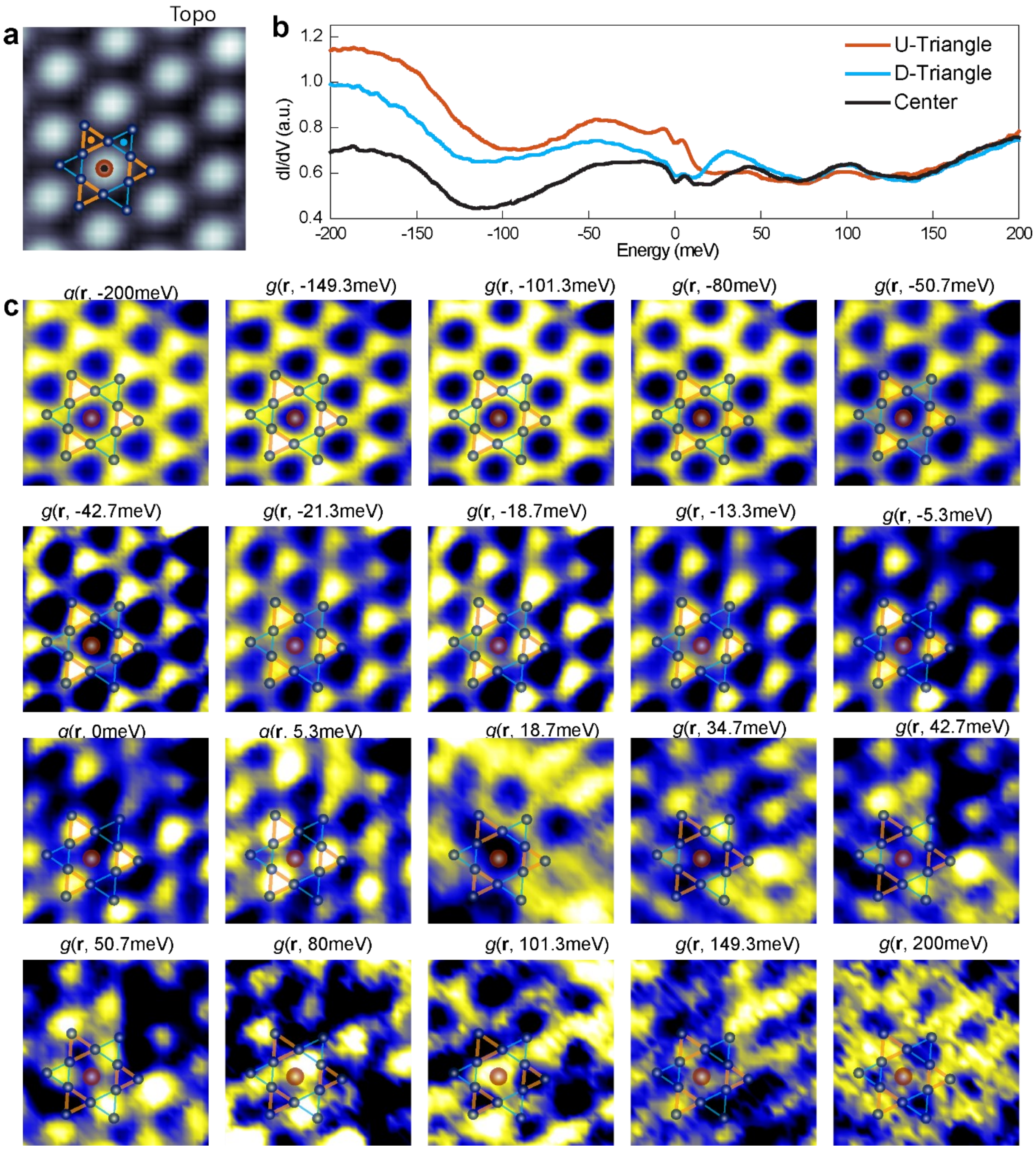

Figure S8. Differential conductance dI/dV mapping of the Fe_3Sn Kagome lattice. a, Topographic STM image of Fe_3Sn Kagome lattice. Setpoint: $V = 0.2$ V, $I = 5.0$ nA. **b**, Typical dI/dV spectra taken at up-triangle, down-triangle, and center sites. **c**, dI/dV maps at the energies indicated. Setpoint: $V = 0.2$ V, $I = 5.0$ nA. The Kagome lattice is overlaid on the topographic image and dI/dV maps.

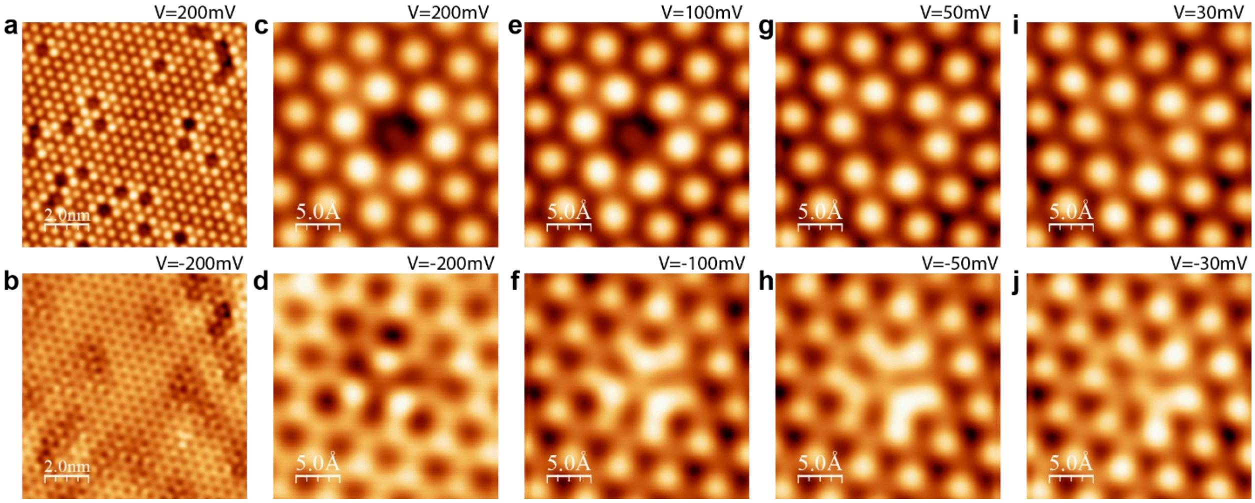

Figure S9. Bias-dependent STM imaging of the Sn vacancy defect on the Kagome lattice. a-b, Large scale STM image of Fe_3Sn layer with Sn vacancies. Setpoint: I = 5.0 nA with V labeled. **c-j**, Closed-up view of a single vacancy defect at the bias indicated. Setpoint: I = 3.0 nA.

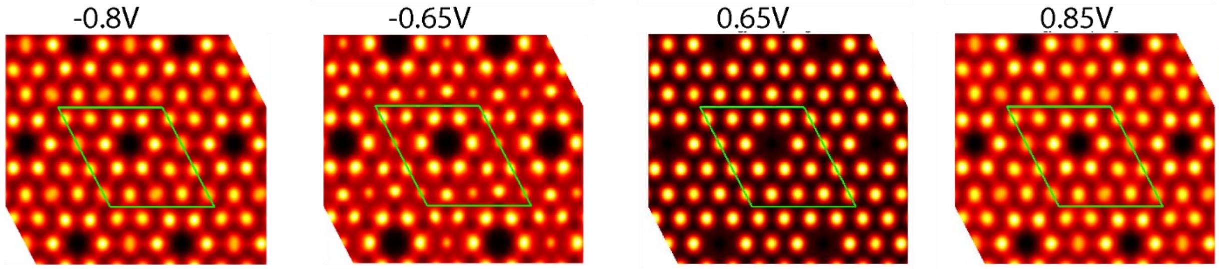

Figure S10. Simulated STM images of the Sn vacancy defect on the Fe_3Sn layer by DFT at the energies indicated.

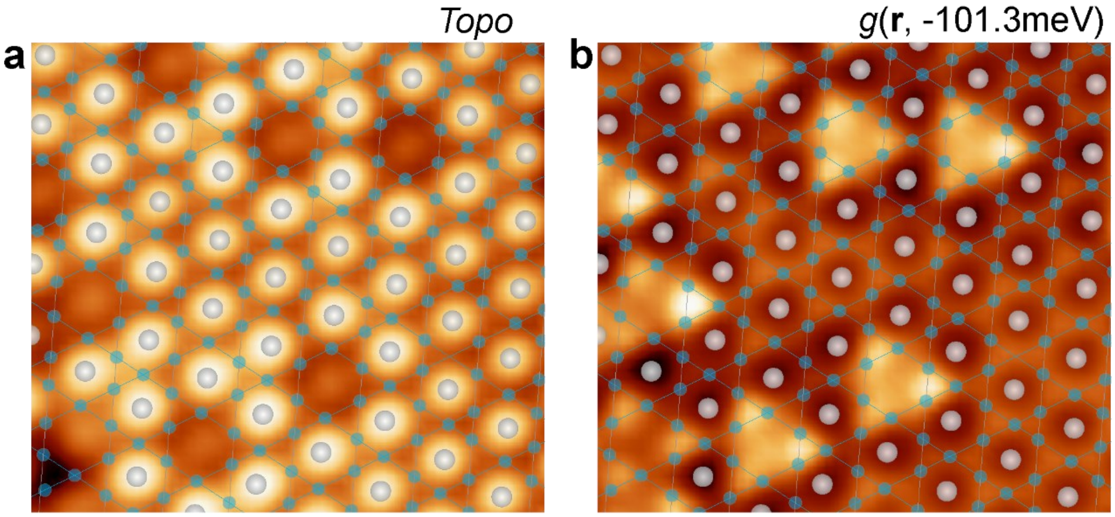

Figure S11. Spatial distribution of the bound states induced by an Sn vacancy on the Kagome layer. **a**, Topographic STM image of Fe_3Sn with vacancies. Lower contrast is observed at the missing Sn atoms. Setpoint: V = 0.2 V, I = 5.0 nA. **b**, Differential conductance dI/dV map g(**r**, -101.3meV). The Kagome lattice is overlaid on both, where gray and blue balls denote Sn and Fe atoms. The contrast at three alternating triangles is enhanced by bound states.

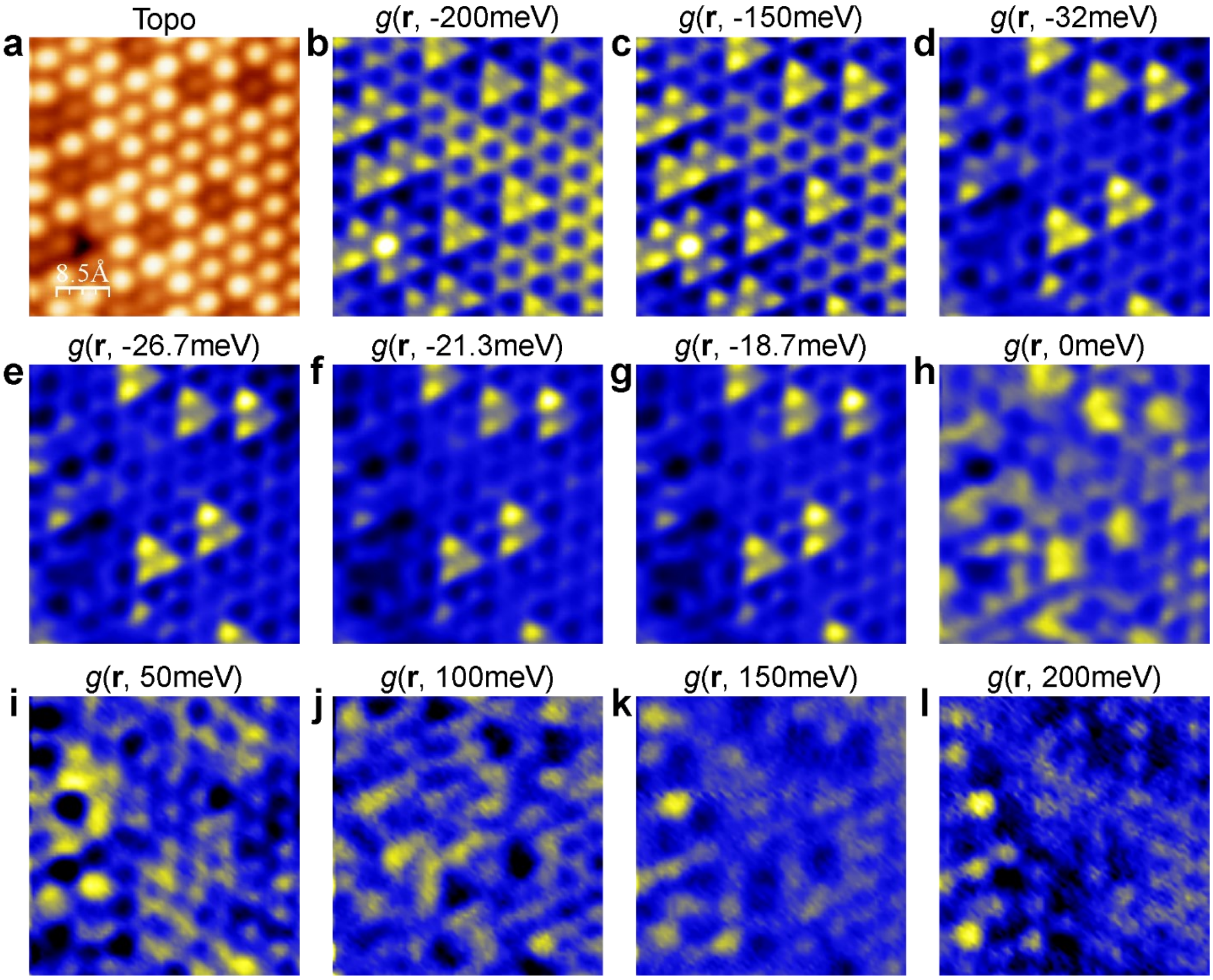

Figure S12. Energy-dependent dI/dV maps of the Fe_3Sn Kagome layer with Sn vacancies. a, Topographic STM image of the Kagome layer with Sn vacancies. Setpoint: V = 0.2 V, I = 5.0 nA. **b-l**, The corresponding dI/dV maps with energy specified, setpoint: V = 0.2 V, I = 5.0 nA, V_{mod} = 3.0 meV.

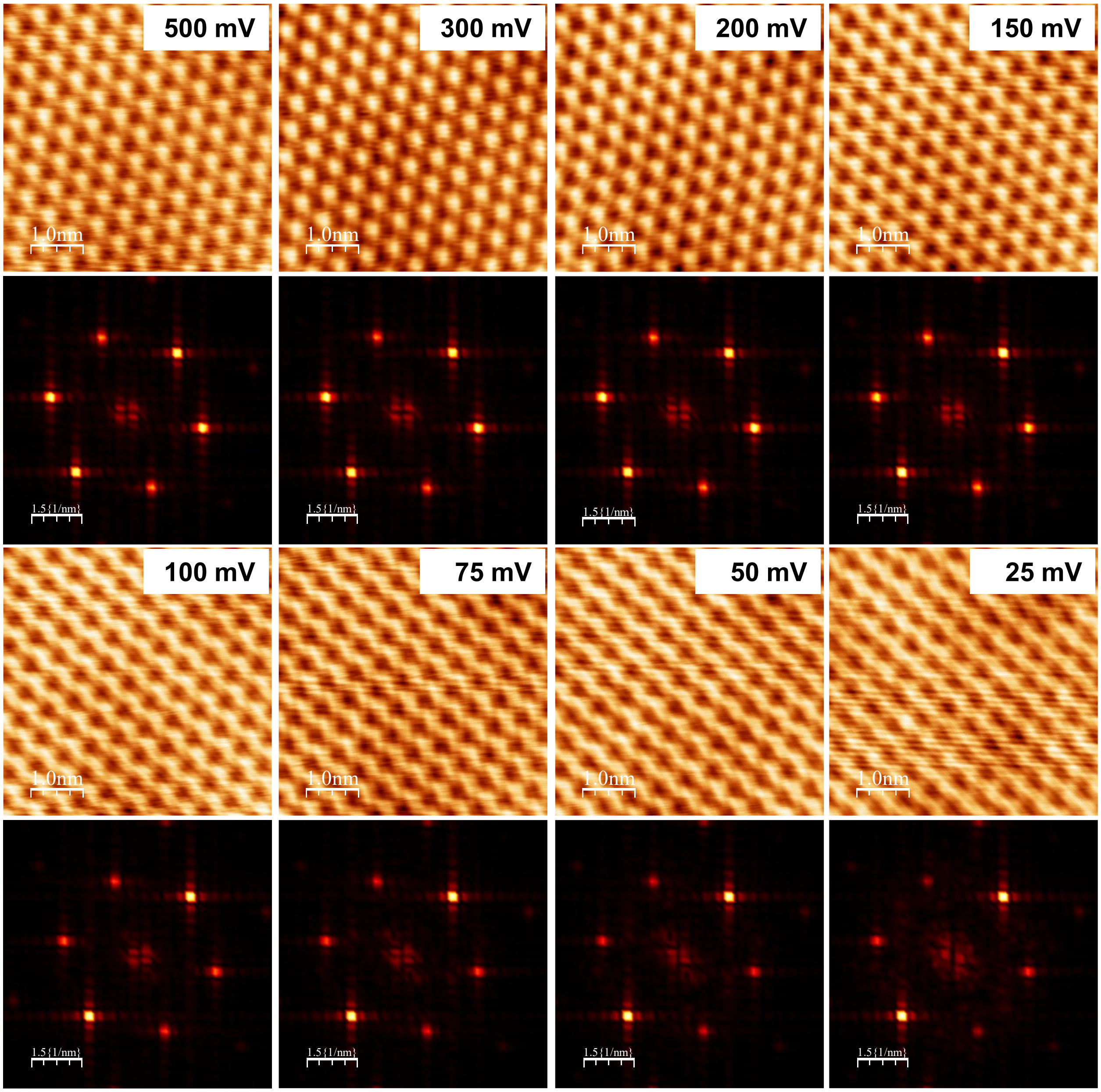

Figure S13 Bias-dependent atomic resolution STM images of the Kagome layer and their corresponding FFTs. The bias is indicated in each image. I_{set} = 500 pA.

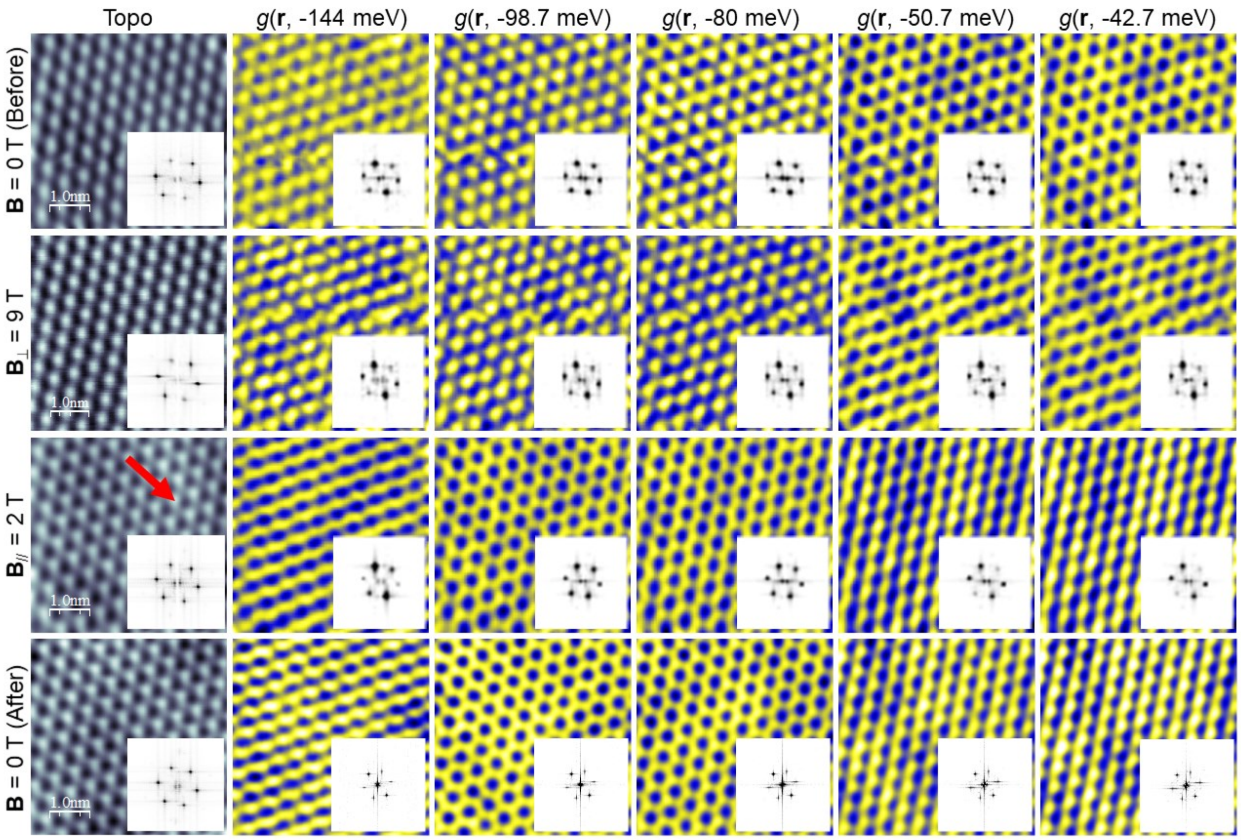

Figure S14. Comparison of dI/dV maps under zero field and vertical and in-plane magnetic fields. dI/dV maps at the energy specified under **B** = 0 T, $\mathbf{B}_{\perp}$ = 9 T, and $\mathbf{B}_{//}$ = 2 T. Setpoint: V = 0.2 V, I = 0.7 nA. The red arrow marks the direction of the in-plane magnetic field B = 2 T

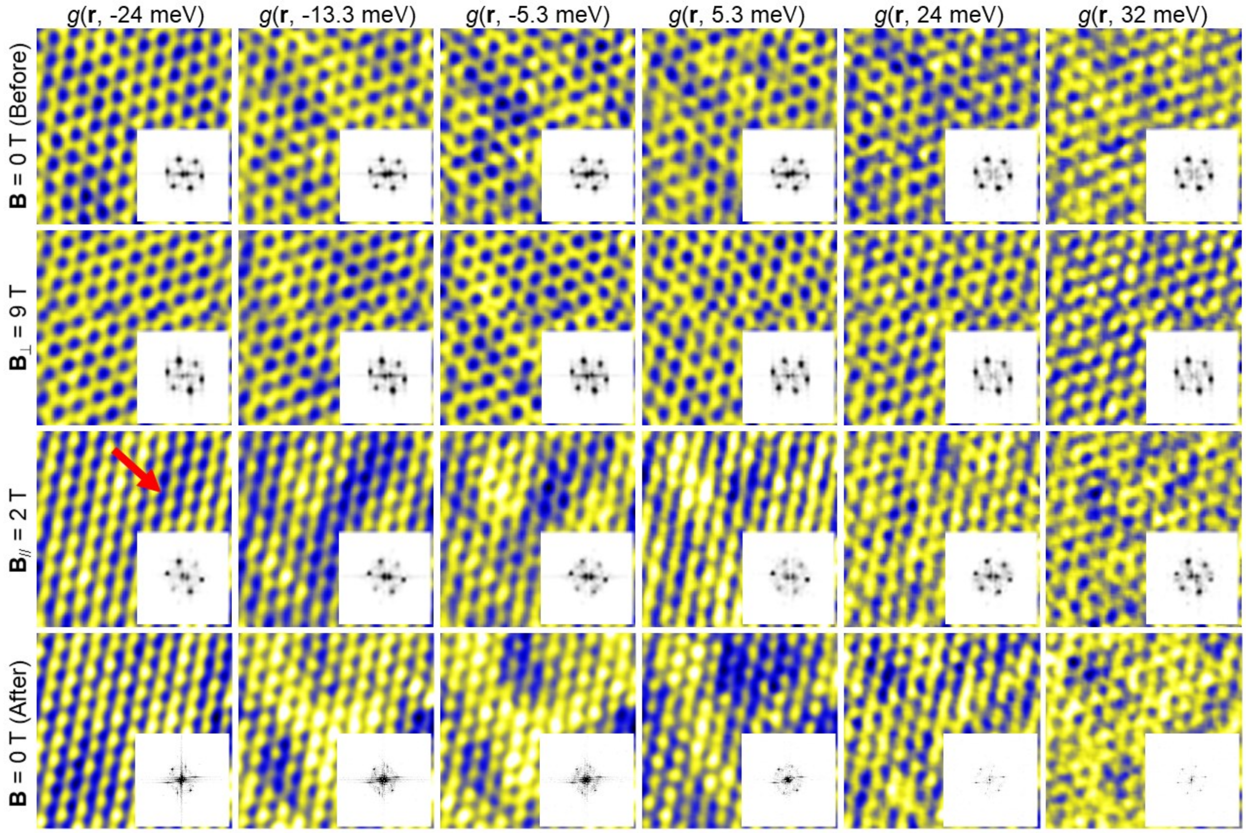

Figure S15. Comparison of dI/dV maps under zero field and vertical and in-plane magnetic fields. dI/dV maps at the energy specified under **B** = 0 T, **B**$_{\perp}$ = 9 T, and **B**$_{//}$ = 2 T. Setpoint: *V* = 0.2 V, *I* = 0.7 nA. The red arrow marks the direction of the in-plane magnetic field B = 2 Tw.

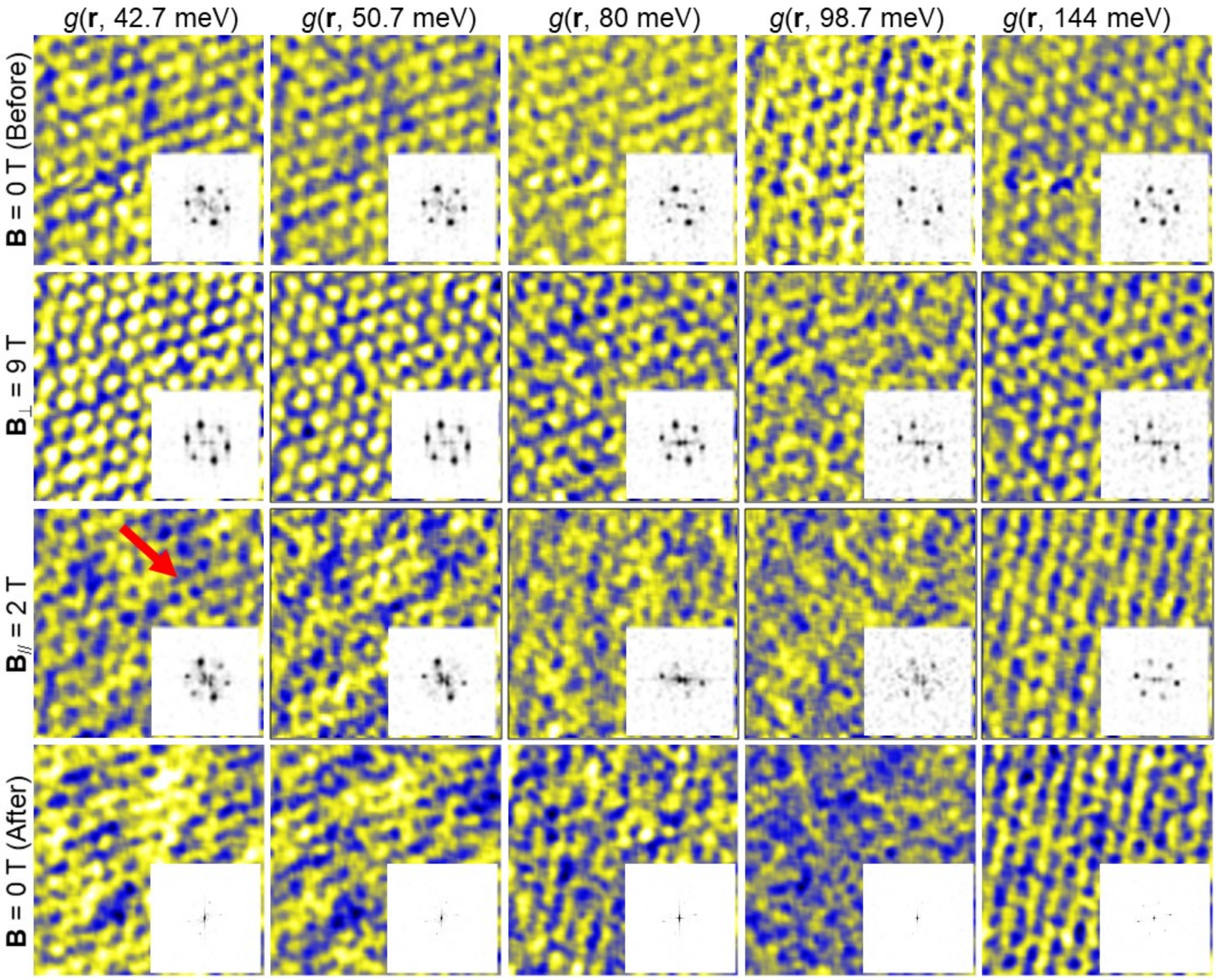

Figure S16. Comparison of dI/dV maps under zero field and vertical and in-plane magnetic fields. dI/dV maps at the energy specified under **B** = 0 T, $\mathbf{B}_{\perp}$ = 9 T, and $\mathbf{B}_{//}$ = 2 T. Setpoint: V = 0.2 V, I = 0.7 nA. The red arrow marks the direction of the in-plane magnetic field B = 2 T.

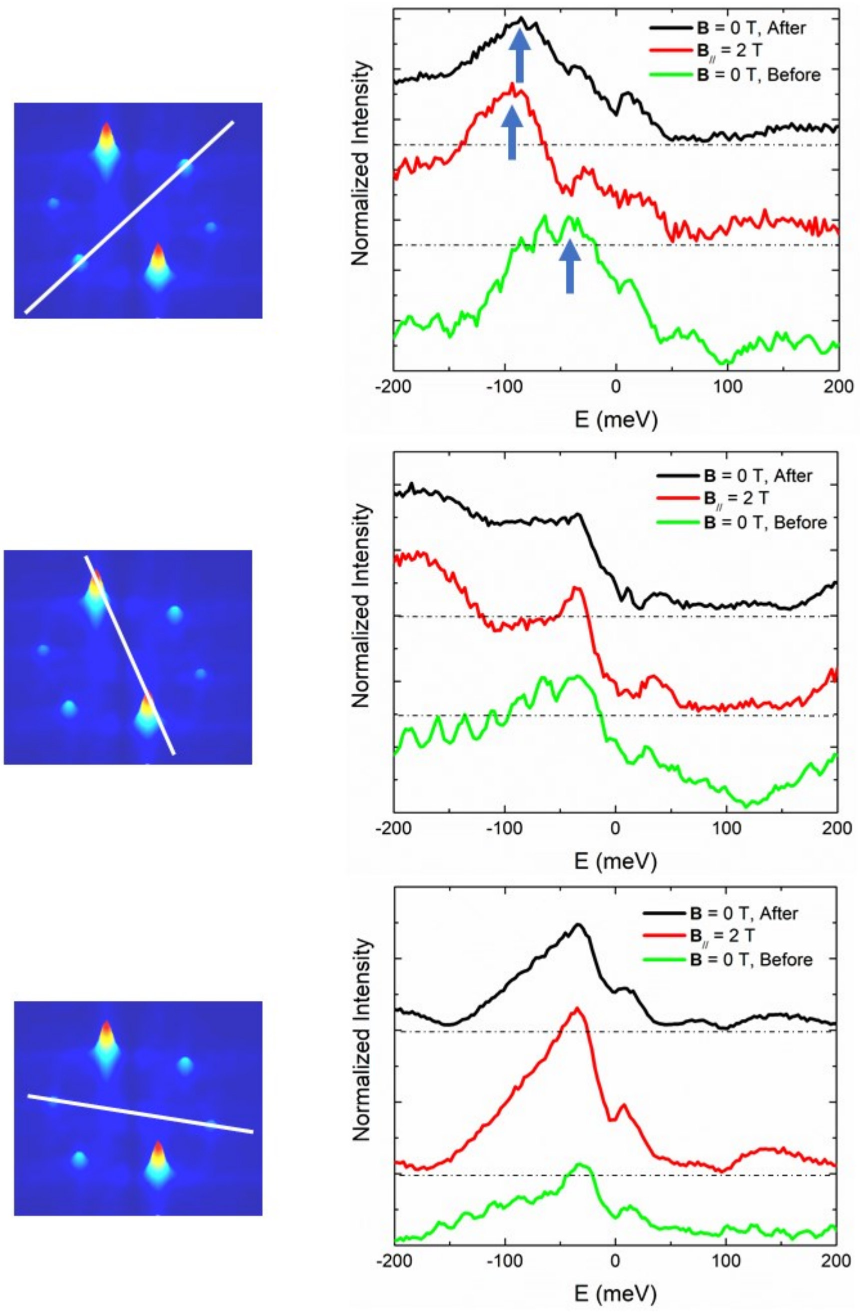

Figure S17. Comparison of FFT peak intensity under zero- and in-plane magnetic fields. FFT peak intensity along the three crystallographic directions as an energy function for all the dI/dV maps at zero and applied parallel fields. The data at $\mathbf{B}_{//}$ = 2 T (red curve) and **B** = 0 T, after (black curve) are shifted vertically. The FFT peak intensity is normalized to their corresponding averaged background intensity.

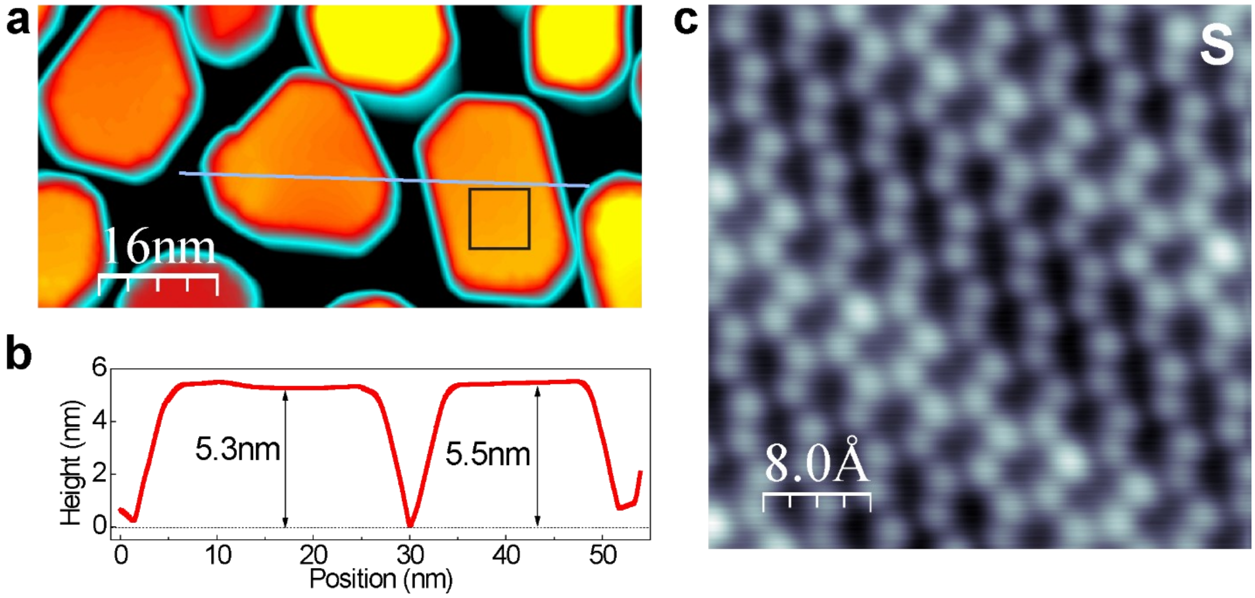

Figure S18. The distorted Sn honeycomb on a strained 5 nm FeSn/STO(111) film. a, Topographic STM image of strained FeSn films epitaxial on $SrTiO_3$(111) substrate, setpoint: V = 1.5 V, I = 10 pA. **b**, Line profile along the cyan line in **a**. **c**, Atomic resolution image taken in the boxed region in **a**, showing the distorted Sn honeycomb and stripes. Setpoint: V = 30 mV, I = 5.0 nA.

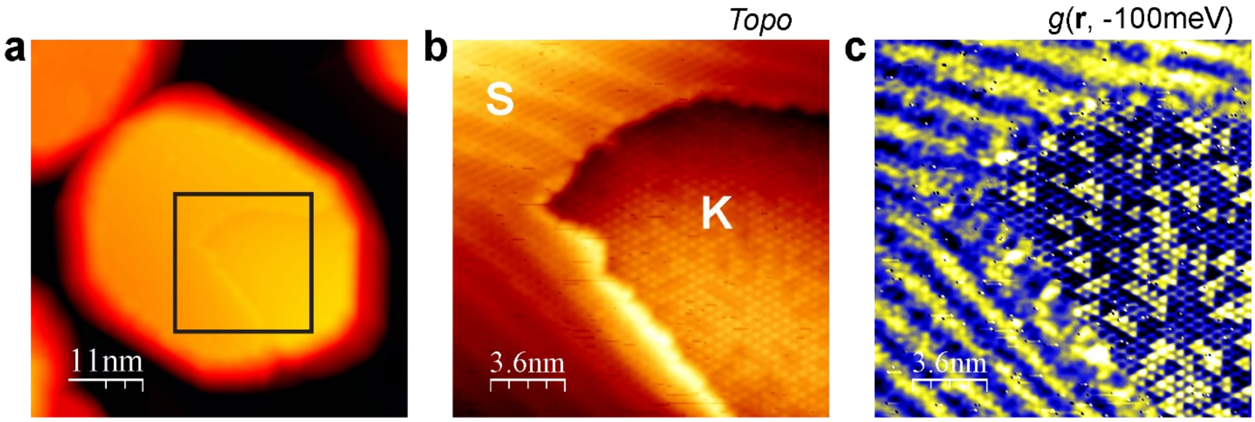

Figure S19. Two terminations in a strained FeSn/STO(111) island. **a**, Topographic STM image of a FeSn island on $SrTiO_3$(111) substrate, setpoint: V = 3.0 V, I = 10 pA. The thickness of the island is 6.1 nm. **b**, Topographic STM image of the boxed region in **a**, showing both the honeycomb (S) and Kagome (K) terminations. Stripes are observed on the S layer. Set point: V = 0.1 V, I = 5.0 nA. **c**, dI/dV map of the region **b**. Setpoint: V = 0.1 V, I = 5.0 nA, V_{mod} = 2 meV. The S layer shows stripe modulations in both topography (**b**) and dI/dV map (**c**), while the K layer exhibits a close-packed structure in topography (**b**) and trimerization in dI/dV map (**c**).
